# No cure for cure models: killed by competing risks

**DOI:** 10.1007/s10985-026-09729-7

**Published:** 2026-09-10

**Authors:** Hein Putter, Per Kragh Andersen

**Affiliations:** 1 Department of Biomedical Data Sciences, Leiden University Medical Center, Leiden, The Netherlands; 2 Section of Biostatistics, University of Copenhagen, Copenhagen, Denmark

**Keywords:** Cure models, Competing risks, Frailties, Mixture models

## Abstract

Cure models have become increasingly popular over the last couple of decades. An appealing element of cure models is the idea that part of the population is immune to the event of interest. We argue that, in medical applications, the use of cure models is limited. Thus, true cure cannot exist if the event of interest includes death, as death is inevitable, and if death is excluded then competing risks must be accounted for. In this case, we find it more natural to model the cumulative incidence of both the event of interest and competing events over time, avoiding unreliable estimates at infinity and using observed data. Further, we point out two more technical difficulties with cure models arising, first, from the fact that cure models rely on identifying plateaus in survival curves at the tail, where data may be sparse and unreliable and, second, the mixture cure model faces issues with practical identifiability of covariate effects in the incidence model (probability of cure), especially alongside proportional hazards assumptions in the latency model (survival model, conditionally on not being cured). We also comment on hybrid extensions of the classical single event cure model that allow for both competing risks and a cured fraction. We support our arguments with real and simulated data. Full data and code are available online.

## Introduction

There is a fast growing literature on statistical cure models within survival analysis and, indeed, there is something appealing about the possibility of modeling the probability of being cured from a disease. Cure models posit the existence of individuals in the population who will never experience the event of interest, no matter how long the follow-up. These individuals are said to be immune or cured.

Two distinct approaches have been developed within this framework, mixture cure models and promotion time cure models. Mixture cure models were first introduced in Boag (1949) and Berkson and Gage (1952), see also Farewell (1982). The basic idea of cure models is the existence of a (latent) part of the population that is immune to the event of interest. For each subject, a latent binary variable *B* is defined, indicating cure (*B=1*) or not (*B=0*). The probability of a subject being cured may depend on covariates *X*, and we define $\pi (X) = P(B = 1 \,|\, X)$ to be the probability of cure, given *X*. Subjects that are cured are immune to the event, so essentially their associated event time *T* is set to *∞ *. Subjects that are not cured (‘uncured’) have conditional hazard $\alpha _u(t \,|\, X)$, and conditional survival function

1 $$\begin{aligned} S_u(t \,|\, X) = P(T > t \,|\, B = 0, X) = \exp \{ - A_u(t \,|\, X) \} = \exp \Bigl \{ - \int _0^t \alpha _u(s \,|\, X) ds \Bigr \}. \end{aligned}$$

The resulting survival function is a mixture of a survival function constantly equal to 1 and the conditional survival function from Eq. (1), leading to

2 $$\begin{aligned} S(t \,|\, X) = \pi (X) + (1 - \pi (X)) S_u(t \,|\, X). \end{aligned}$$

The mixture cure model is fully defined after specifying probability models for the cure probabilities *π (X)*, the so-called *incidence model*, and for the conditional hazards $\alpha _u(t \,|\, X)$, the *latency model* (e.g., Peng and Taylor 2014; Amico and van Keilegom 2018). The standard model used for *π (X)* is a logistic regression model, where

3 $$\begin{aligned} \text {logit}(\pi (X)) = \gamma _0 + \gamma ^\top X. \end{aligned}$$

For $\alpha _u(t \,|\, X)$, the standard model is a proportional hazards model

4 $$\begin{aligned} \alpha _u(t \,|\, X) = \alpha _{u0}(t) \exp (\beta ^\top X) \end{aligned}$$

and the resulting model will be referred to as the proportional hazards mixture cure model (PH mixture cure model).

One way to view the PH mixture cure model is as a special case of a *univariate frailty model*. Frailty models include a multiplicative random effect *Z*, here *Z=1-B*, called a frailty term, such that $\alpha (t \,|\,X, Z) = Z \alpha _u(t \,|\,X)$, where $\alpha _u(t \,|\,X)$ is the conditional hazard, given *Z=1*. Usually it is assumed that *Z* has unit mean, but if instead *Z* is taken to be binary and *P(Z=1)* is allowed to depend on *X*, this leads to a marginal survival function following Eq. (2).

The second type of models used to model cure are the promotion time cure models. They are motivated by models for the growth of cancer cells (Yakovlev et al. 1996) and lead to a survival function of the form

5 $$\begin{aligned} P(T>t) = S(t) = \exp (-\theta Q(t)). \end{aligned}$$

Here, *Q*(*t*) is the distribution function for a certain *proper* random variable and *θ >0*. It follows that

$$\begin{aligned} \lim _{t\rightarrow \infty }S(t)=\exp (-\theta )>0, \end{aligned}$$

i.e., *T* is an *improper* random variable with $P(T=\infty )=\exp (-\theta )$, corresponding to the cured fraction, *π *.

Covariates *X* may be included in Eq. (5), e.g., by letting $\theta (X)=\exp (\gamma _0+\gamma ^\top X)$ and leaving *Q*(*t*) independent of *X*. This is a proportional hazards model for *T*, however, with a bounded cumulative hazard. The proportionality assumption may be relaxed by introducing covariates into *Q*(*t*), e.g., by assuming

$$\begin{aligned} 1-Q(t\mid X)=(1-Q_0(t))^{\exp (\beta ^\top X)}, \end{aligned}$$

with a certain (proper) ‘baseline distribution function’ $Q_0(t)$.

For reviews on cure models we refer to Peng and Taylor (2014) and Amico and van Keilegom (2018), and the books Maller and Zhou (1996) and Peng and Yu (1996).

The objective of this paper is to argue that we find the application of cure models quite limited in a medical context. This is because, in humans, cure in the sense of having zero probability to experience the event of interest simply cannot exist if the event of interest contains death of any cause. If, on the other hand, the event of interest does not contain death of any cause, then death or all causes of death not included in the definition of the event of interest must be competing risks and should be considered as such. Further, we point out two more technical difficulties arising when analyzing cure models. First, cure is identified at the very tail of the survival curve by identifying ‘plateaus’ and ensuring ‘sufficient follow-up’ (to be discussed in more detail in Sect. 3). However, the tail is precisely the part of the follow-up where least information about the underlying hazard is available. The second difficulty is specific to the mixture cure model and questions the practical identifiability of the covariate effects in the incidence model in combination with the proportional hazards assumption in the latency model. These objections apply to the classical single event cure model as introduced above. Hybrid extensions of this model that allow for both competing risks and a cured fraction have been studied by, e.g., Basu and Tiwari (2010) and Conlon et al. (2013)—both in a Bayesian framework—and by Beesley and Taylor (2019) who considered a more general illness-death model.

The paper is structured as follows. Section 2 argues that there is no such thing as cure in humans when death of any cause is part of the event in question, and that in other cases competing risks should be taken into account in a proper way. Section 3 gives an example where the existence of plateaus can easily be (falsely) concluded when in fact there is no cure present, even after formal tests for the existence of plateaus and sufficiency of follow-up have been performed. Section 4 shows that, specifically in mixture cure models, violation of the proportional hazards assumption in the latency model may lead to substantial bias in the estimation of the covariate effects in the incidence model. We conclude with a brief discussion in Sect. 5, putting our reservations in perspective.

## Cure models versus competing risks

Cure models for events that include overall death (e.g. Ibrahim et al. 2001 who studied recurrence-free survival in malignant melanoma), to us, make little sense: There is no cure for overall death. Furthermore, studying illustrative examples used in the biostatistical literature on cure models, it appears that studies of recurrence in cancer are common. For instance, Amico and van Keilegom (2018) exemplified cure models using a study on distant metastases in breast cancer and Peng and Taylor (2014) and Li and Taylor (2002) using a study on local recurrence in tonsil cancer. In such studies, ‘cure’ means ‘no recurrence’ and, as a consequence, in the mixture cure model *π (X)* represents the probability of *never* having a recurrence. Thus, cure is a ‘no event’ and attempting to model that nothing is happening, to us, seems less natural than modeling the actual occurrence of events. This point of view also immediately leads to the question ‘if the patient does not have a recurrence, then what happens to him or her instead?’—a question that further leads to studying *competing risks*.

In cure models, the arguments are based on a survival curve that describes, at any time *t* (including *t=∞ *), the probability of not having had the event. This survival function is known to be incorrect in the presence of competing risks (e.g., Andersen et al. 2012) because competing events are treated as censored observations. We would argue that one should rather model the probability over time that the event has, indeed, occurred and do this jointly with modeling the occurrence over time of competing events, i.e., primarily death (from other causes). The value at *t=∞ * of the cumulative incidence of competing causes is the probability that the event never happens, but instead of attempting to set up a model for the value at infinity (which is hard to get a handle on with censored data—the core of the problem with cure models, and which is incorrectly estimated by a Kaplan–Meier estimator in the presence of competing risks), the suggested approach would model the cumulative incidence of competing causes over time based on actual observations of occurrences of the competing event.

### Example: recurrence after bone marrow transplantation

**Fig. 1:**
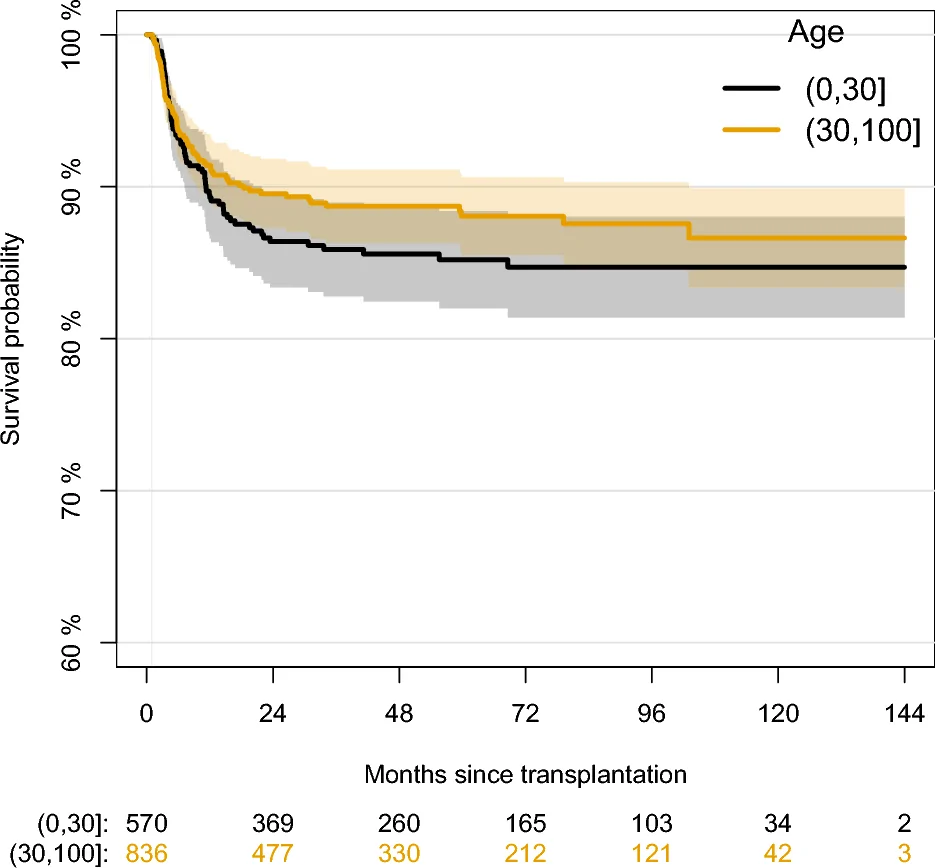
Kaplan–Meier survival curves for relapse for 1406 patients with AML

Andersen and Pohar Perme (2008) studied the disease course after bone marrow transplantation for 2009 patients with acute leukemia. The data are available from Andersen and Ravn (2023). Here, we will study the subgroup of patients with acute myeloid leukemia (AML, *n=1406*) and a single covariate, *X*, which is the indicator of age above 30 years (*X=1* for 836 patients). Among patients with AML, 154 patients experienced a relapse (the event of interest) and 343 died in remission (the competing cause). Figure 1 shows the Kaplan–Meier curves for relapse, incorrectly censoring for death in remission. The curves both indicate a plateau after *≈ 120* months, slightly lower for the younger age category (values at 120 months are 0.847 and 0.866, respectively). Fitting a cure model could, therefore, seem appropriate.

A PH mixture cure model yields a coefficient $\widehat{\beta }=0.048 (\text {SE}=0.150)$, suggesting an insignificantly higher relapse rate for the older age group *among patients who will eventually relapse*, the latency part of the cure model. The incidence part yields an estimated intercept of $\widehat{\gamma }_0=1.656\; (\text {SE}=0.085)$ and a coefficient $\widehat{\gamma }_1=0.243\;(\text {SE}=0.148)$ for age above 30 years, suggesting an (insignificantly) higher probability of cure for the older age group. The predicted cure probabilities from this logistic incidence model (0.840 and 0.870, respectively) are well compatible with the Kaplan–Meier plateaus and do suggest that older patients tend to more likely be cured.

**Fig. 2:**
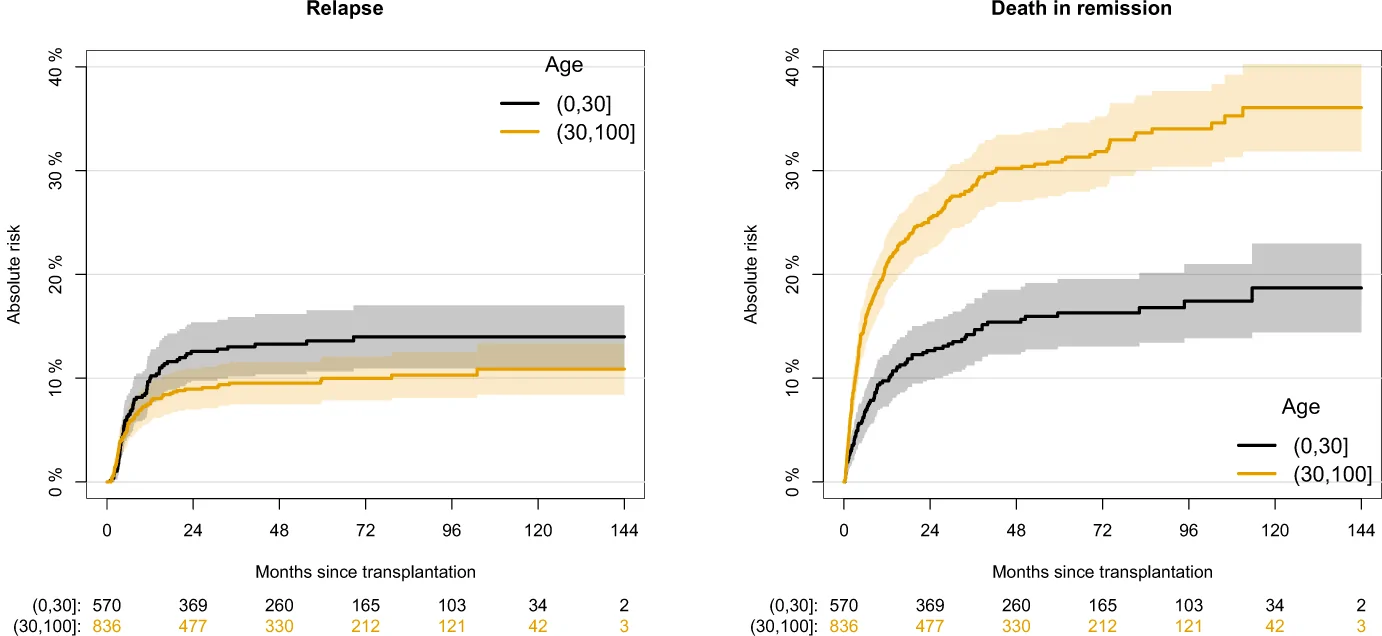
Aalen–Johansen cumulative incidence curves for relapse and for death in remission for 1406 patients with AML

Instead of this approach to the question of the relationship between relapse and age for patients with AML, we would recommend a standard competing risks approach. This would entail, first, estimating the cumulative incidence of relapse and that of death in remission using the Aalen–Johansen estimator, see Fig. 2.

**Fig. 3:**
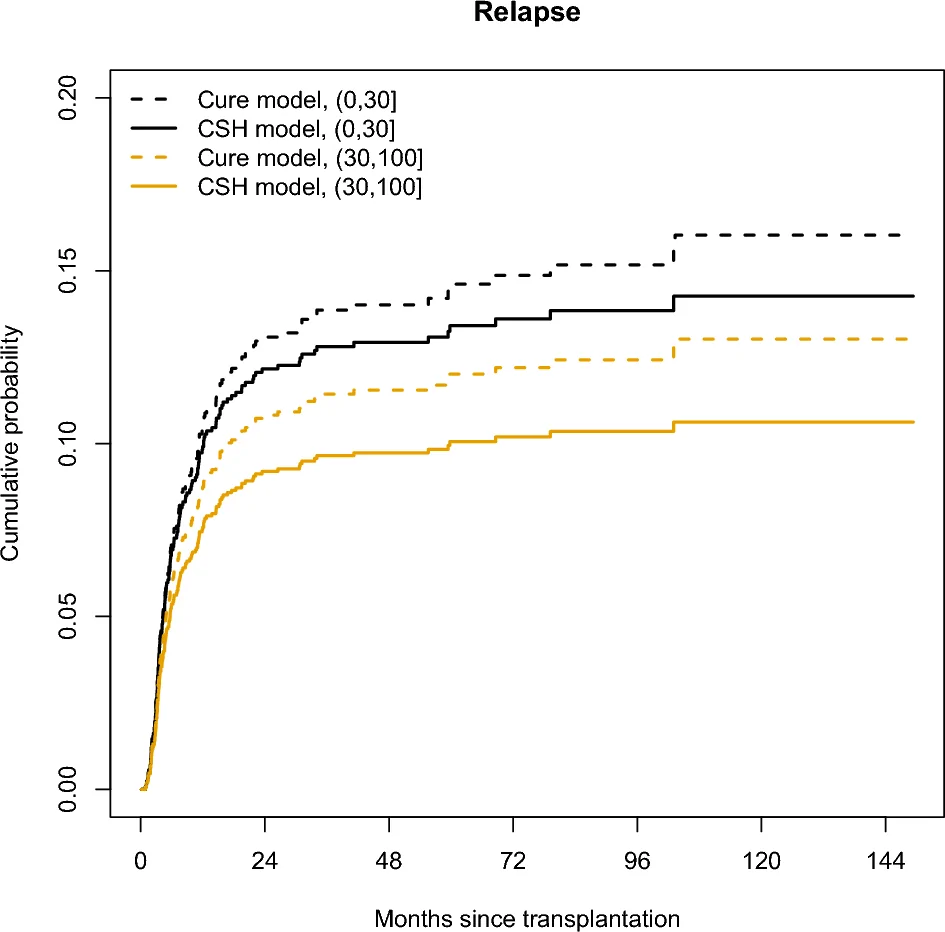
Model-based cumulative incidence curves for relapse for younger and older patients with AML (*n=1406*), based on the cure model and on the proportional cause-specific hazards (CSH) model

This figure suggests a higher risk of relapse for the younger age group and an appreciably higher risk of death in remission for the older age group. Continuing the competing risks analysis, it appears that older patients have a non-significantly lower cause-specific hazard for relapse ($\log (\text {HR})=-0.203, \text {SE}=0.116$) and a highly significantly higher cause-specific hazard for death in remission ($\log (\text {HR})=0.774, \text {SE}=0.124$). Figure 3 shows estimates of the cumulative probability of relapse for both age groups, based on the cure model and on the cause-specific hazards models. The non-parametric cumulative incidence probabilities of relapse at the end of follow-up, as shown in Fig. 2, were estimated at 10.9% and 14.0% for younger and older age groups, respectively. Clearly, the cause-specific hazard based models in Fig. 3 are in line with these, while those based on the cure models are over-estimated, because they ignored competing risks.

*□ *

We will, therefore, argue that the competing risks analysis is preferable for several reasons. First, it correctly estimates the relapse probabilities in the real world where patients may die in remission, whereas the cure model estimates these probabilities in a hypothetical world where the competing risk is not present. Second, it provides information about what may happen to patients who do not experience relapse and, finally, it leads to the more plausible conclusion that older patients do not tend to be cured from relapse, but rather that they tend to die in remission.

Models that combine cure with competing risks have been discussed. However, in the model of Chen et al. (2020) there is still a cured fraction of patients who will never experience neither the event of interest, nor a competing event and we would argue that this ‘cured fraction’ of patients is simply those who die from other causes without having experienced any of the two events. Also, Xue et al. (2022) in a context of in-hospital mortality (the event of interest) and discharge (‘observed cure’) discussed the relationship between a promotion time cure model and a competing risks model for the event of interest with observed cure serving as a competing event. Finally, Nicolaie et al. (2019) studied so-called ‘vertical modeling’ of competing risks data with a cured fraction of subjects who would never experience any of the competing events. As mentioned in the Introduction, there are also extensions of the classical single event cure model in which a fraction of the subjects cannot experience the event of interest (e.g., recurrence), but where everyone is at risk for the competing event (e.g., death in remission) in the context of a competing risks model (Basu and Tiwari 2010), or an illness-death model (Conlon et al. 2013; Beesley and Taylor 2019). We discuss the latter model in relation to a standard competing risks analysis in Sect. 5.

## Identifiability of cure plateaus

The presence of a latent sub-population that is cured of the disease of interest must be based on the tail of the time-to-event distribution. The most important practical limitation for establishing the presence of cure is that the tail of the time-to-event distribution is precisely the part of the distribution that is most difficult to estimate. Towards the end of follow-up, the numbers at risk may be small and the estimates of the survival probabilities imprecise. The follow-up distribution is finite per definition. Looking at the tail of the Kaplan–Meier estimates and trying to say something about the behaviour of the survival function at infinity seems wishful thinking.

We will illustrate the difficulties with a simulated data set. Full code in R for all data generation and analyses performed is available on github at https://github.com/survival-lumc/CureModels. The true underlying data generation mechanism will be revealed towards the end of this section. Based on the simulated data we follow theory from Chapter 4 of the classical book of Maller and Zhou (1996) to first test for the presence of a cure fraction, and subsequently test for sufficiency of follow-up to establish and reliably estimate this cure fraction.

**Fig. 4:**
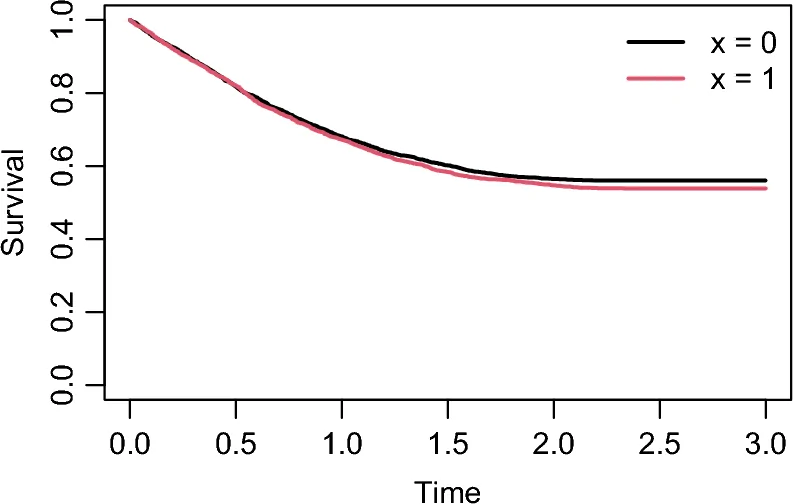
Kaplan–Meier survival curves for two values of a binary covariate suggesting a plateau

**Fig. 5:**
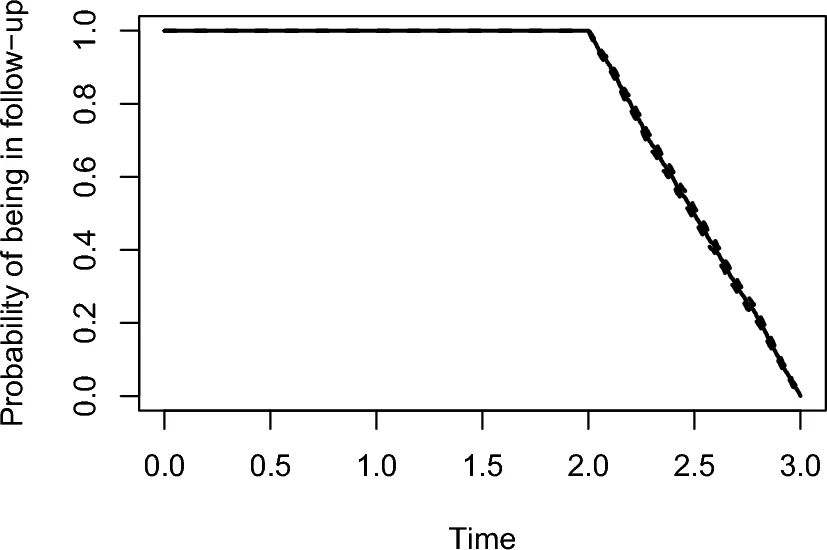
Kaplan–Meier estimate of the censoring distribution (assumed to be common to *x=0* and *x=1*)

Figure 4 shows Kaplan–Meier survival curves for two values of a binary covariate *x*, based on 2500 observations from a true underlying survival curve $S(t \,|\, x=0)$ (black) and 2500 observations from a true underlying survival curve $S(t \,|\, x=1)$ (red). Both estimated survival curves seem to suggest a plateau, either with identical or different values around 55 to 60%. Figure 5 shows a plot of the Kaplan–Meier estimate for the censoring distribution, assumed (correctly) to be common to *x=0* and *x=1*. The plot strongly suggests a uniform censoring distribution between 2 and 3, which is in fact the true underlying censoring distribution.

Maller and Zhou (1996) describe a strategy of first testing whether or not there is evidence of a plateau (or, in this case, two plateaus), and second testing whether there is sufficient follow-up to establish the presence of these plateaus. In Appendix 1, see also https://github.com/survival-lumc/CureModels, we elaborate on these tests and apply them to the data underlying Figs. 4 and 5. The short summary to report here is that 1) the plateaus for *x=0* and *x=1* are significantly different from 0, and 2) there is evidence of sufficient follow-up to establish the presence of these plateaus.

Fitting a mixture cure model using the R package {intsurv} (Wang et al. 2021) leads to estimated plateaus of 0.560 for *x=0* and 0.539 for *x=1*. Results are in complete accordance with the plateaus seen by the Kaplan–Meiers, so together with the overwhelming evidence that both for *x=0* and *x=1* there is a plateau, and that we have sufficient follow-up to establish this and estimate its proportion, we can be quite confident about these results.

**Fig. 6:**
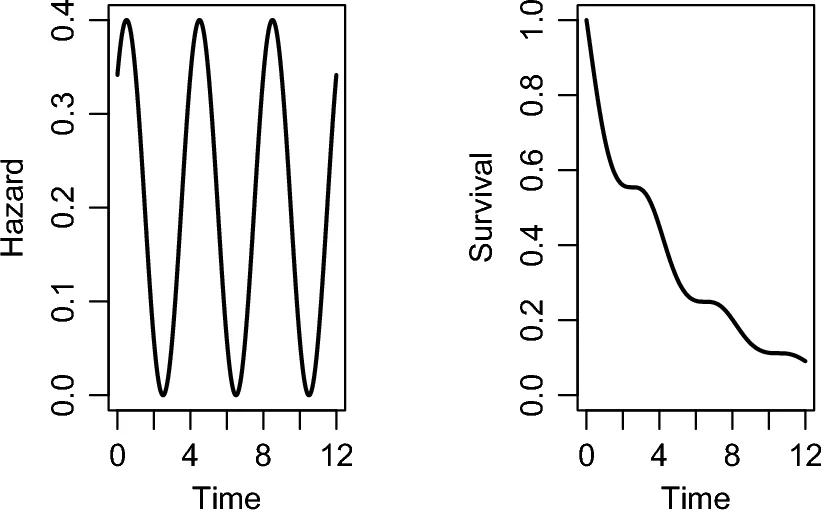
The true underlying hazard (left) and the true underlying survival function (right)

Or can we? Time to reveal the true nature of the data that we just generated. The true time-to-event distributions for *x=0* and *x=1* are the same. Figure 6 shows the true underlying hazard (left) and the true underlying survival function (right). The hazard is a sine wave function, periodically close to 0, around *t=2.5, 6.5, 10.5* etcetera. The true underlying survival function actually goes to zero as time goes to infinity, so in reality there is no proportion cured at all.

**Fig. 7:**
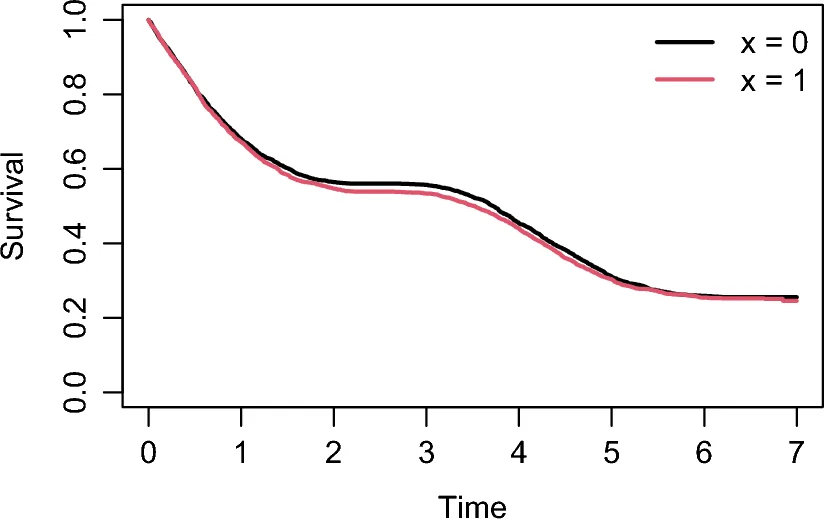
Kaplan–Meier survival curves for x = 0 and x = 1, uniform censoring on (6, 7)

If we use the same uncensored data leading to Fig. 4 with uniform censoring on (2, 3), and instead change the censoring interval to (6, 7), this leads to the Kaplan–Meier estimates for *x=0* and *x=1* as shown in Fig. 7. This time a plateau around 25% is visible. Checking the existence of a non-zero plateau and sufficiency of follow-up using the methods of Maller and Zhou (1996) again leads us to be confident about these results. Fitting a cure model to this data gives estimated plateaus of 0.253 for *x=0* and 0.248 for *x=1*. Again we are misled about the existence of a plateau.

One may argue that the survival curve on the right in Fig. 6 looks quite contrived. However, our main point with this example is to illustrate that, in reality, nothing is known beyond the last time of censoring. Furthermore, it is not uncommon to see Kaplan–Meier curves that go down at regular time points and are constant in between. Two recent examples from the New England Journal of Medicine are Fig. 2a from Mellinghoff et al. (2023) and Figs. 1a and b from Xi and Henry (2024). Both studies have progression-free survival as endpoint, where progression is assessed at regular times pre-specified by the study protocol. The particular form of the Kaplan–Meier estimate is in fact caused by ignoring *interval censoring*; when progression has not been established at one assessment but has been established at the next assessment, the event, progression, has in fact taken place in the interval between these assessment. The absence of progression could be interpreted as being cured from the disease, so from the perspective of cure modelers a plateau in the Kaplan–Meier survival curve could be indicative of the presence of a cure fraction. Especially later in the study, when successive assessments tend to become less frequent, it is quite possible that absence of events (because they are not assessed) may be mistaken for the presence of a plateau.

## Identifiability of cure probability models

As mentioned in Sect. 1, the proportional hazards mixture cure model, Eq. (2), is as a special case of a univariate frailty model. It is well known that without covariates, the univariate frailty model is not identifiable, but it *is* identifiable when $\alpha _u(t \,|\,x)$ follows a proportional hazards model (Elbers and Ridder 1982). The underlying reason for this is that the presence of the frailty term leads to a violation of the proportional hazards assumption for the marginal hazard. There is no way to distinguish between certain violations of the proportional hazards assumption and the presence of frailty (Balan and Putter 2020). We now show that similar issues play a role for the PH mixture cure model.

For simplicity we consider the simple situation of a single covariate *x*, but now consider also possible non-PH models for $\alpha _u(t \,|\, x)$, defined as $\alpha _u(t \,|\, x) = \alpha _{u0}(t) \exp (\beta (t) x)$, with *β (t)* not necessarily constant. The survival function for given *x* is then given by (cf. Eq. (2)):

$$\begin{aligned} S(t \,|\, x) = \pi (x) + (1 - \pi (x)) \exp \Bigl \{ - \int _0^t \alpha _{u0}(s) \exp (\beta (s) x) ds \Bigr \}, \end{aligned}$$

with *π (x)* the probability of cure as before, and $\exp \Bigl \{ - \int _0^t \alpha _{u0}(s) \exp (\beta (s) x) ds \Bigr \}$ the conditional survival function (conditional on not being cured). Note that the limit for $t \rightarrow \infty$ of $S(t \,|\, x)$ equals *π (x)*, but only if $\int _0^\infty \alpha _{u0}(s) \exp (\beta (s) x) ds = \infty$.

We define $\alpha (t \,|\, x)$ to be the resulting marginal (so not conditional on uncure) hazard given *x*, given by the derivative with respect to *t* of $-\log (S(t \,|\, x))$. Calculating this derivative gives

$$\begin{aligned} \alpha (t \,|\, x) = \frac{(1 - \pi (x)) \exp \Bigl \{ - \int _0^t \alpha _{u0}(s) \exp (\beta (s) x) ds \Bigr \}}{\pi (x) + (1 - \pi (x)) \exp \Bigl \{ - \int _0^t \alpha _{u0}(s) \exp (\beta (s) x) ds \Bigr \}} \alpha _{u0}(t) \exp (\beta (t) x). \end{aligned}$$

We recognize the phenomenon known from frailty models, where the marginal hazard is progressively dragged down with respect to the conditional hazard (Balan and Putter 2020).

It is not hard to find conditional PH and non-PH models, respectively, that lead to the same marginal hazards $\alpha (t\,|\, x=0)$ and $\alpha (t\,|\, x=1)$, but with different incidences $\pi (0), \pi (1)$. Details are given in Appendix 2. Thus, the PH model with $\alpha _{u0}(t) \equiv 1$, $e^\beta = 2$, *π (0)=1/2*, and *π (1)=2/3*, has the same marginal hazards as the non-PH model with $\alpha _{u0}(t) \equiv 1$, ${\beta }(t) = \log \left( \frac{2}{1 + e^{2t}} \right)$, *π (0)=1/2*, and *π (1)=1/3*. In the former (PH) case, the odds ratio for cure (*x=1* vs. *x=0*) is 2, while in the latter (non-PH) case it is 1/2, i.e., it is reversed.

We conducted a simulation, based on *M=1000* replications with data sets of size *n=1000* generated from PH and non-PH mixture cure models with a binary covariate *x* with *P(X = 0) = P(X = 1) = 0.5*, $\text {logit}(\pi (x)) = - \log (2) x$, $\alpha _u(t \,|\,x = 0) \equiv 1$, and $\alpha _u(t \,|\,x = 1) = \frac{2}{(1 + e^{2t})^\lambda }$. The variable *λ * was varied from $\lambda = 0, 0.2, \ldots , 0.8, 1$. The model for *λ = 0* follows the proportional hazards mixture cure model which has an odds ratio of 1/2, while the model for *λ = 1* follows a non-proportional hazards mixture cure model shown to be equivalent to a PH mixture cure model with an odds ratio of 2. Hence, for *λ =1* and a binary covariate we would expect that, falsely assuming conditional proportional hazards as software for mixture cure models does, this would lead to an inversely estimated odds ratio of 2.

**Fig. 8:**
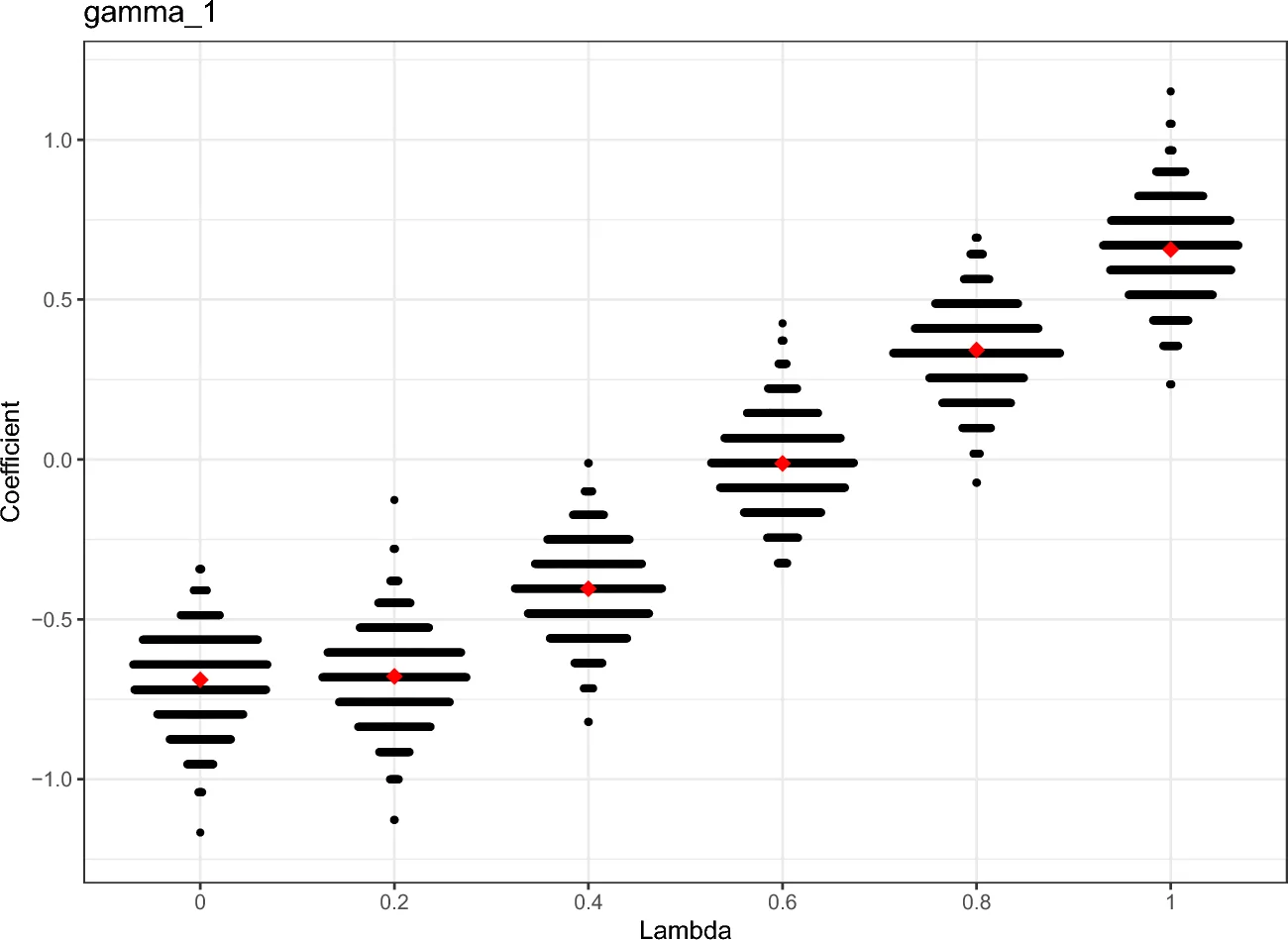
Estimated log odds ratios for *x* of the probability of being cured, *x* binary with *P(X = 0) = P(X = 1) = 0.5*

Figure 8 shows scatter plots of the estimated log odds ratios for *x* of the probability of being cured for the different values of $\lambda = 0, 0.2, \ldots , 0.8, 1$, when *X* is taken to be binary. We see that for *λ = 0*, the estimated log odds ratios are indeed centered around the true value of *-łog (2)*. When *λ * increases, the mean estimated log odds ratio increase, ultimately leading to an average value of *+łog (2)* for *λ = 1*.

Obviously, one of the (PH or non-PH) models is misspecified, and one may argue that what we have demonstrated is just the effect of fitting an incorrect model to the data. However, in a practical situation, it is challenging to evaluate the fit of a conditional model given a latent variable and the simulation then shows that fitting the standard PH mixture cure latency model may lead to incorrect inference for the incidence model.

## Discussion

Cure models have an intuitive appeal in that it seems reasonable to think that part of the population is immune to the event of interest. They have a long history in statistics, going back three quarters of a century (Boag 1949; Berkson and Gage 1952), also finding use in more complex time-to-event settings like multi-state models, through the mover-stayer models (Goodman 1961). There is a tight connection with frailty models, which we have exploited in Sect. 4. If the frailty distribution has a point mass at 0, like in the compound Poisson frailty distributions, then this corresponds to a mixture cure model. The difference in typical applications of such frailty models is that the frailty distribution is usually not depending on covariates.

The illness-death cure model of Beesley and Taylor (2019) does, indeed, meet some of our criticism against the classical single event cure model. This is because it allows for a cured fraction in relation to recurrence and still lets all subjects be at risk for death without recurrence. However, in comparison with such a model we still think that a standard competing risks model has a number of advantages. The cure model makes the assumption that some subjects will *never* experience recurrence—an assumption that we find unnecessarily strong when based on, by definition, finite follow-up. On the contrary, in a standard competing risks analysis, it would be possible to ascertain covariates associated with a *low* rate of recurrence and, further, no assumption of the existence of a latent variable would be needed — such an analysis would simply be based on the data as they were actually observed. The interpretation of covariate effects in the competing risks model is also more simple, as they are not conditional on a latent variable, and so is goodness-of-fit assessment. As a final point, we mention that the identifiability issues discussed for the classical cure model in Sect. 4 will likely carry over to the illness-death cure model.

The mathematical results of identifiability (e.g., Li et al. 2001, later generalized by Hanin and Huang 2014), include the sufficient condition that $\tau _F < \tau _G$, with $\tau _F$ and $\tau _G$ the end of the domains of the time-to-event and censoring distributions, respectively. If we want to interpret *π (x)* as the probability of cure, then it follows that $\lim _{t \rightarrow \infty } S_u(t \,|\,x) = 0$ must hold. (If $\lim _{t \rightarrow \infty } S_u(t \,|\,x) > 0$, then the probability of cure is not *π (x)* but $\pi (x) + (1 - \pi (x)) \lim _{t \rightarrow \infty } S_u(t \,|\,x)$.) Together with the unavoidable fact that $\tau _G$ is always finite by design, which in turn implies that $\tau _F$ is finite, we would have $S_u(\tau _F \,|\,x) = 0$, which can only happen if $H_u(\tau _F \,|\,x) = \infty$, so the hazard $h_u(t \,|\,x)$ is either infinite or tending to infinity for some $0 < t \le \tau _F$. This makes for quite unnatural hazard models. Hardly any of the popular parametric models used in survival analysis have bounded support, an exception being the uniform distribution on $[0,\tau _F]$. Moreover, this fact is never used, not in any simulation study on cure models that we have seen and not in software like {intsurv} or {smcure} (Cai et al. 2022).

As emphasized in previous sections, cure models make little sense if the event of interest includes all-cause mortality. In such situations, however, the concept of ‘statistical cure’ may be useful. This is based on models for the excess hazard compared to population-based life-tables and was studied by Pocock et al. (1982) and more recently by Yu et al. (2004), Lambert et al. (2007), and Eloranta et al. (2014). Here, ‘statistical cure’ is obtained at the time where the mortality rate for the patients reaches that of the standard population. A concept close to that of statistical cure was discussed by (van Houwelingen and Putter 2012, Section10.4) under the heading of ‘relative survival guarantee’.

We have relied on existing statistical software in R to fit semi-parametric mixture cure models, in particular {intsurv} and {smcure}. In the simulated data of Sects. 3 and 4, {intsurv} and {smcure} gave identical results, but in the analysis with real data of Sect. 2 results obtained by {smcure} and {intsurv} were quite different, with {smcure} results looking quite unreliable. We therefore report results by {intsurv} throughout this paper. Results obtained by {smcure} can be found through the online material at https://github.com/survival-lumc/CureModels.

In conclusion, in spite of their intuitive appeal and interesting mathematical challenges, we believe that cure models should rarely be a method of choice for survival analysis in medical applications. For all-cause mortality, they are inappropriate and, in other situations, they build on a marginal survival curve that is incorrect in the presence of competing risks and attempt to model the tail of the distribution for which censoring prevents reliable observation. In such situations, well-established methods for competing risks could do the job instead.

## Figures and Tables

**Fig. 9:**
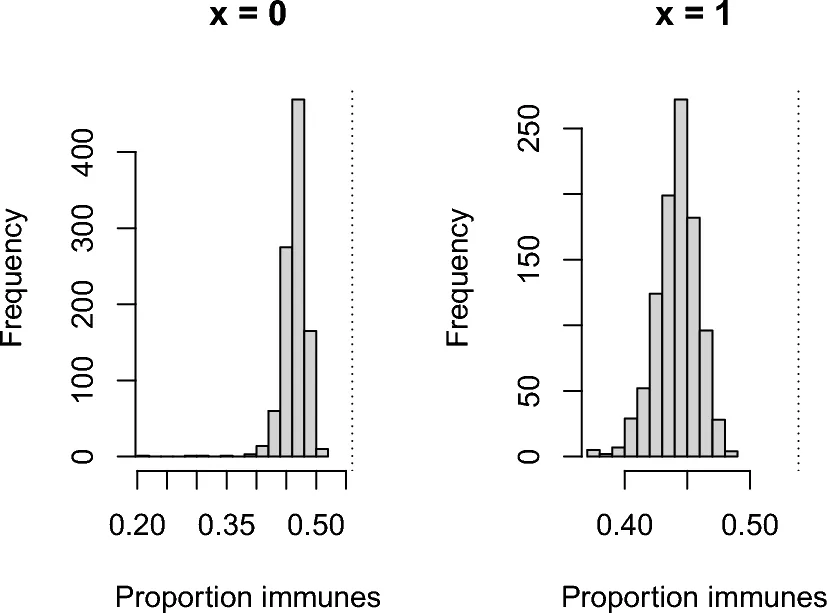
Histogram of the plateau estimates under $H_{01}$, for *x=0* (left) and *x=1* (right)

**Fig. 10:**
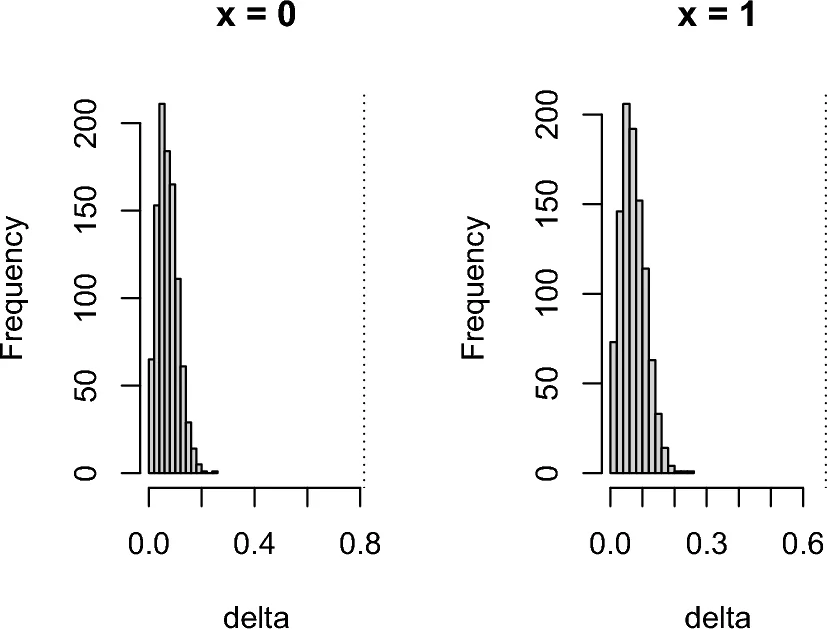
Histogram of $\delta _n$ under $H_{02}^c$, for *x=0* (left) and *x=1* (right)

**Fig. 11:**
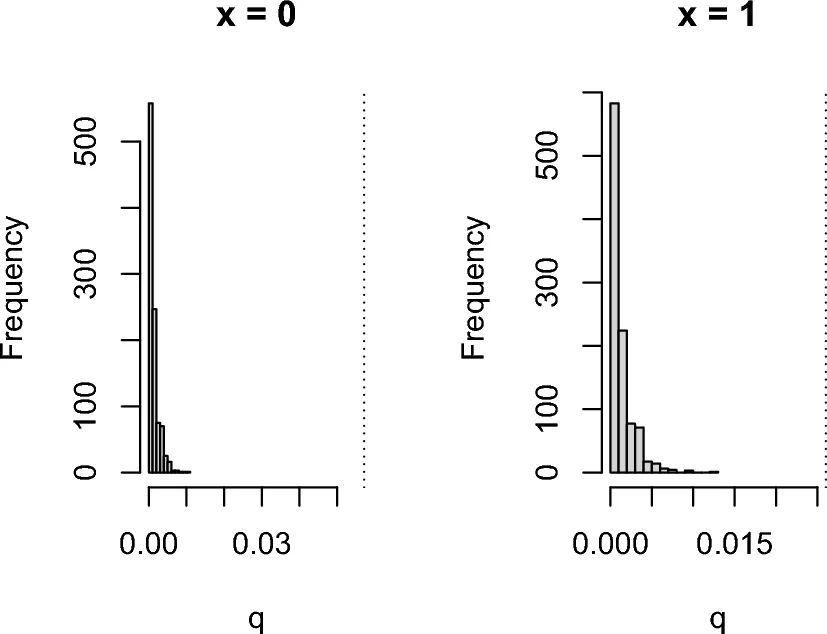
Histogram of $q_n$ under $H_{02}^c$, for *x=0* (left) and *x=1* (right)

## Appendix 1

Here, we give more details in connection with analysis of the simulated data in Sect. 3. The first question to address is whether there is evidence of a plateau. The first thing then is to estimate the proportion of immunes in each group, the natural estimator being the Kaplan–Meier evaluated at the last observed time point, irrespective of whether it is an event or censoring time point, which we will denote by $\hat{p}_n$. These Kaplan–Meier estimates are 0.560 for *x=0* and 0.539 for *x=1*. In what follows in this section, we will follow the theory of Maller and Zhou (1996) in testing for the presence of a plateau and sufficiency of follow-up. The theory is non-parametric, so we will apply it separately for *x=0* and *x=1*. Maller and Zhou (1996) start out by testing $H_{01}: F(\tau _G) = 1$, in Sect. 4.2, where *F(· )* is the cumulative distribution function of the time-to-event, and $\tau _G$ is the end of follow-up.

An alternative way of expressing this null hypothesis is $H_{01}: S(\tau _G) = 0$. If $H_{01}$ is rejected, this is seen as evidence for a plateau, and we would move on to test sufficiency of follow-up, to be addressed later.

The proposed test for $H_{01}$ is to reject $H_{01}$ if $\hat{p}_n > c_{0.05}$, where $\hat{p}_n$ as before is the Kaplan–Meier evaluated at the last observed time point (event or censored), and $c_{0.05}$ is the 5th percentile of the distribution of $\hat{p}_n$ calculated under $H_{01}$. To calculate the distribution of $\hat{p}_n$ under $H_{01}$ is difficult in general, it will depend on the unknown distributions *F* and *G* of the censoring and survival times. Maller and Zhou (1996) approximate this distribution under a range of distributions of *F* and *G*, the percentiles of which are reported in a number of tables. We will not rely on their published tables, since their assumed censoring distributions are different from ours, but follow the reasoning of their procedure, and calculate the percentiles by simulation, separately under *x=0* and *x=1* (since we are going to test $H_{01}$ in each of the subgroups). We take the censoring distributions (correctly) to be uniform on (2, 3), and, following Maller and Zhou (1996) the event distribution exponential with rates to be estimated from the observed data (separately for *x=0* and *x=1*). The assumption of underlying exponential distributions may well be incorrect, but recall that we have to work from $H_{01}$ anyway.

We used 1000 simulated data sets of size *n=2500* (same as the actual data) with *x=0* and with *x=1*, generating event data from exponential distributions with rates estimated from the data (0.256 for *x=0* and 0.274 for *x=1*) and applying independent uniform censoring on (2, 3). Within each of the simulated data sets we calculated and recorded $\hat{p}_n$. Figure 9 shows histograms of these simulated $\hat{p}_n$ under $H_{01}$, separately for *x=0* (left) and *x=1* (right), along with the estimated values from the actual data indicated with vertical lines. All simulated $\hat{p}_n$ values under $H_{01}$ are larger than the estimated $\hat{p}_n$ in the data both for *x=0* and for *x=1*. It is clear that we would reject $H_{01}$, both for *x=0* and *x=1*, with *p<0.001*.

That leaves the second question: is there sufficient follow-up to establish the presence of these plateaus? This is addressed in Section 4.3 of Maller and Zhou (1996), by defining the second hypothesis $H_{02}: \tau _F \le \tau _G$, and its complement $H_{02}^c: \tau _F > \tau _G$, where $\tau _F$ and $\tau _G$ are the right-hand side of the supports of *F*, the event-time distribution and of *G*, the censoring distribution. Maller and Zhou (1996) argue that we need to ‘use the information in the sample regarding the magnitude of $\tau _G - \tau _F$, which is effectively a measure of how far the large censored lifetimes (the potential immunes) lie from the main mass of the susceptibles’ lifetime’. Evidence for sufficient follow-up is to be found in the difference between the largest failure time $t_{\text {max}}$ and the largest uncensored failure time $t_{\text {max}}^*$. This leads to a test statistic for the null hypothesis $H_{02}^c$ of the form $\delta _n = t_{\text {max}} - t_{\text {max}}^*$. Maller and Zhou (1996) also consider $q_n = N_n / n$, with $N_n$ the number of uncensored $t_i$ in $(2 t_{\text {max}}^* - t_{\text {max}}, t_{\text {max}}]$. The test will then be to reject $H_{02}^c$ in favour of $H_{02}$ if $\delta _n$ or $q_n$ exceed certain critical values, and to accept $H_{02}^c$ otherwise. These critical value then depend on the distribution of the test statistics under $H_{02}^c$. Again, these distributions can be approximated by simulation. For *x=0* we see in our data that $t_{\text {max}}$, $t_{\text {max}}^*$, $\delta _n$ and $q_n$ are given by 2.999, 2.184, 0.815, and 0.057, respectively. For *x=1*, the observed $t_{\text {max}}$, $t_{\text {max}}^*$ and $q_n$ are given by 3.000, 2.329, 0.671, and 0.026, respectively.

We use the same setting to approximate the null distributions (under $H_{02}^c$) for $\delta _n$ and $q_n$, so in essence the same generated data sets were used as before, but this time the test statistics $\delta _n$ and $q_n$ were calculated and stored for each generated data sets (separately for *x=0* and *x=1*). The resulting histograms are shown in Figs. 10 and 11. It is clear that both for *x=0* and *x=1*, the estimated values obtained from the data are completely outside the histogram of the values generated under $H_{02}^c$, again leading us to the clear conclusion that follow-up is sufficient to establish and reliably estimate the plateaus.

## Appendix 2

Here we show how to find conditional PH and non-PH models, respectively, that lead to the same marginal hazards $\alpha (t\,|\, x=0)$ and $\alpha (t\,|\, x=1)$, but with different incidences $\pi (0), \pi (1)$. Denote

$$\begin{aligned} A_{ux}(t) = \int _0^t \alpha _{u0}(s) \exp (\beta (s) x) ds \end{aligned}$$

to be the conditional cumulative hazard for given frailty and given value of *x*. We will fix $\pi _0$, $\pi _1$, *β * and $\alpha _{u0}(t)$ for the conditional proportional hazards model, where the cure model is correctly specified, and seek to find an alternative choice (referred to as “non-PH setting”) $\tilde{\pi }_0$, $\tilde{\pi }_1$, $\tilde{\beta }(t)$ and $\tilde{\alpha }_{u0}(t)$ for which both $S(t \,|\, x=0)$ and $S(t \,|\, x=1)$ are identical. For given $\tilde{\beta }(t)$ and $\tilde{\alpha }_{u0}(t)$, define $\tilde{A}_{u0}(t) = \int _0^t \tilde{\alpha }_{u0}(s) ds$ and $\tilde{A}_{u1}(t) = \int _0^t \tilde{\alpha }_{u0}(s) \exp (\tilde{\beta }(s)) ds$. Also define $\text {OR} = \frac{\pi _1}{1-\pi _1} / \frac{\pi _0}{1-\pi _0}$ to be the odds ratio of cure for *x=1* compared to *x=0*, and similarly $\widetilde{\text {OR}} = \frac{\tilde{\pi }_1}{1-\tilde{\pi }_1} / \frac{\tilde{\pi }_0}{1-\tilde{\pi }_0}$. We want to choose $\tilde{\pi }_0$, $\tilde{\pi }_1$ in such a way that the odds ratio $\widetilde{\text {OR}} \not = \text {OR}$.

Equating $S(t \,|\, x)$ for the PH and non-PH settings for *x=0*, gives

$$\begin{aligned} \pi _0 + (1-\pi _0) \exp (-A_{u0}(t)) = \tilde{\pi }_0 + (1-\tilde{\pi }_0) \exp (-\tilde{A}_{u0}(t)). \end{aligned}$$

Suppose that we fix $\pi _0$, $\alpha _0(t)$ and $\tilde{\pi }_0$. Solving for $\tilde{A}_{u0}(t)$ then yields

$$\begin{aligned} \tilde{A}_{u0}(t) = -\log (\pi _0 - \tilde{\pi }_0 + (1 - \pi _0) \exp (-A_{u0}(t)) + \log (1-\tilde{\pi }_0), \end{aligned}$$

from which we get

6 $$\begin{aligned} \tilde{\alpha }_{u0}(t) &= \frac{(1 - \pi _0) \exp (-A_{u0}(t))}{\pi _0 - \tilde{\pi }_0 + (1 - \pi _0) \exp (-A_{u0}(t))}\nonumber \\ \alpha _{u0}(t) &= \alpha _{u0}(t) \left( 1 + \frac{\pi _0 - \tilde{\pi }_0}{1 - \pi _0} \exp (A_{u0}(t)) \right) ^{-1}. \end{aligned}$$

In the same way we get for *x=1*:

7 $$\begin{aligned} \tilde{\alpha }_{u1}(t) &= \frac{(1 - \pi _1) \exp (-A_{u1}(t))}{\pi _1 - \tilde{\pi }_1 + (1 - \pi _1) \exp (-A_{u1}(t))}\nonumber \\ \alpha _{u1}(t) &= \alpha _{u1}(t) \left( 1 + \frac{\pi _1 - \tilde{\pi }_1}{1 - \pi _1} \exp (A_{u1}(t)) \right) ^{-1}. \end{aligned}$$

For the PH setting we take $\pi _0 = 1/2$, $\pi _1 = 2/3$, leading to an odds ratio for cure (*x=1* wrt *x=0*) of 2 $( = \text {OR})$, and for the non-PH setting we set $\widetilde{\text {OR}} = 1/2$ (a *reversed effect* of *x* on the probability of cure). This is achieved by setting $\tilde{\pi }_0 = 1/2$ and $\tilde{\pi }_1 = 1/3$. By deducing $\tilde{\alpha }_{u0}(t)$ and $\tilde{\alpha }_{u1}(t)$, from Eqs. (6) and (7), we find $\tilde{\beta }(t)$ by the relation $\widetilde{\text {HR}}(t) = \exp (\tilde{\beta }(t)) = \frac{\tilde{\alpha }_{u1}(t)}{\tilde{\alpha }_{u0}(t)}$. For the specific choice of $\alpha _{u0}(t) \equiv 1$ and $e^\beta = 2$, and $\tilde{\pi }_0 = 1/2$, $\tilde{\pi }_1 = 1/3$, we obtain

8 $$\begin{aligned} \tilde{\alpha }_{u0}(t) \equiv 1, \quad \tilde{\alpha }_{u1}(t) = \frac{2}{1 + e^{2t}},\quad \tilde{\beta }(t) = \log \left( \frac{2}{1 + e^{2t}} \right) . \end{aligned}$$
